# Automatic measurement and prediction of Chinese Grown Pigs weight using multilayer perceptron neural networks

**DOI:** 10.1038/s41598-023-28433-2

**Published:** 2023-02-13

**Authors:** Obiajulu Emenike Ositanwosu, Qiong Huang, Yun Liang, Chukwunonso H. Nwokoye

**Affiliations:** 1 College of Mathematics and Informatics, South China Agricultural University, Guangzhou, 510642 China; 2 Guangzhou Key Laboratory of Intelligent Agriculture, South China Agricultural University, Guangzhou, 510642 China; 3 Department of Computer Science, Nnamdi Azikiwe University, P.M.B. 5025, Awka, Nigeria; 4 ABM College of Health and Technology, Toronto, Canada

**Keywords:** Computational science, Computer science, Information technology

## Abstract

The knowledge of body size/weight is necessary for the general growth enhancement of swine as well as for making informed decisions that concern their health, productivity, and yield. Therefore, this work aims to automate the collection of pigs’ body parameters using images from Kinect V2 cameras, and the development of Multilayer Perceptron Neural Network (MLP NN) models to predict their weight. The dataset obtained using 3D light depth cameras contains 9980 pigs across the S21 and S23 breeds, and then grouped into 70:15:15 training, testing, and validation sets, respectively. Initially, two MLP models were built and evaluations revealed that model 1 outperformed model 2 in predicting pig weights, with root mean squared error (RMSE) values of 5.5 and 6.0 respectively. Moreover, employing a normalized dataset, two new models (3 and 4) were developed and trained. Subsequently, models 2, 3, and 4 performed significantly better with a RMSE value of 5.29 compared to model 1, which has a RMSE value of 6.95. Model 3 produced an intriguing discovery i.e. accurate forecasting of pig weights using just two characteristics, age and abdominal circumference, and other error values show corresponding results

## Introduction

Presently, the growing need for livestock production has necessitated the development of an effective answer to issues surrounding the measurement of animal bodies. Collecting data on animal physical characteristics is very essential because the understanding of these variables can reflect developmental progress, productive capacity, genetic features^1^, fat deposition, and energy consumption^2^. Researchers have established that several key behavioral attributes have been demonstrated to possess enough heritability in such a manner that genetic choices aimed at altering them will indeed be achievable^3^. Most observations meant for gathering the necessary body data are performed manually with measuring tapes, rulers, and measuring scales. Apart from the fact that this method is time-consuming, it is also seen to be dangerous and harmful to the animals, that may be subjected to infections or distress as a result of this type of monitoring^4^. Considering that animals are prone to shifting and/or discontinuing a particular activity at the sight of an investigator, this enormous time commitment may potentially lead to false findings. More importantly, manual measuring is rarely used on commercial farms, and is limited to body weight measures during the growth period in some phases. Building an automated measuring approach to examine phenotypic traits, however, has previously been regarded as a constraint in biology^5^. With all these, the development of a human-independent, automated strategy to address the aforementioned issues is necessary.

**Figure 1:**
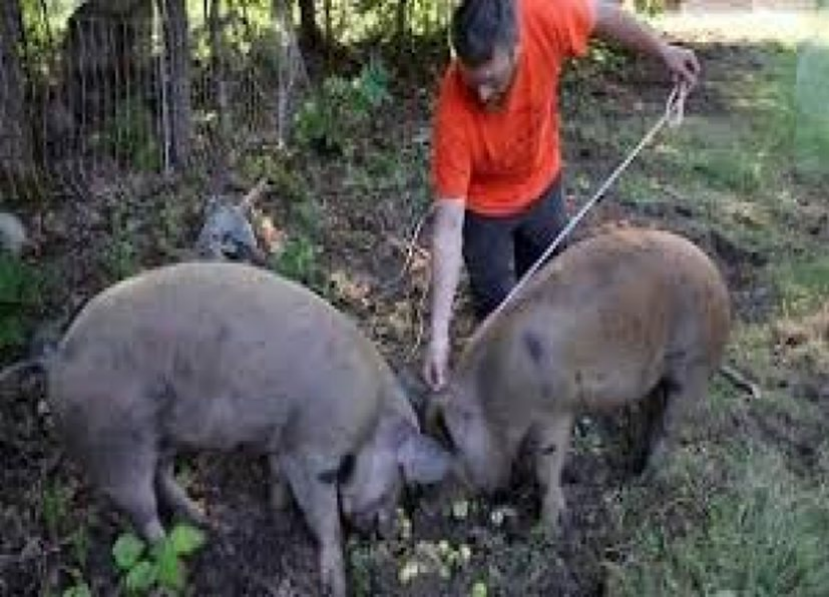
Manual measurement of Pigs length.

**Figure 2:**
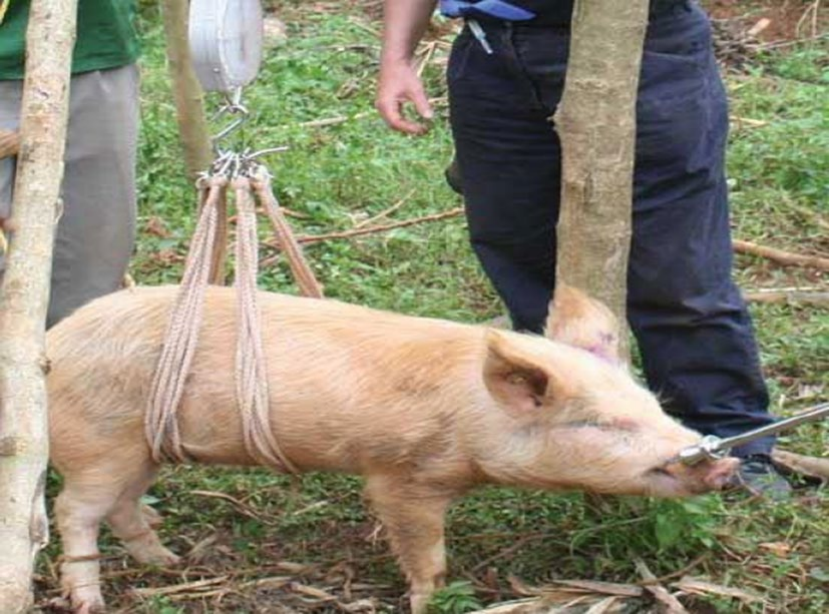
Manual measurement of Pigs weight.

Pigs are quite important in today's society. Aside from hens and cows, it is clear that pigs were the primary experimental animal for automated behavior monitoring. Pork is indeed the most prominent meat globally, with roughly 1 billion pigs raised annually and over 120 million tonnes of meat produced^6^. Animal health has an impact on both production and welfare, but there are a plethora of concerns when it comes to huge farms. Modern detection methods for pig diseases and treatments may include human intervention in the form of inspections, as well as daily/quarterly checkups by ranch staff or veterinary professionals. On manual measurement, Fig. 1 and 2 show the obtaining of the physical parameters of pigs. Furthermore, note that bias may be added in the assessment of behavioral/clinical indications and symptoms, resulting in potentially unreliable renderings of pen operations^7^. Most deterioration in health commences with a shift in the level of physical activity and/or a reduction in meal and liquid intake, according to Taylor^8^, signs that are tough to identify in a cursory pen visit. In certain circumstances, manually identifying diseases that persist can be demanding and difficult. As two or more stockmen are required for manual measurement, commercial farms require an alternative that is less threatening, less stressful, and less disturbing to the animals, thus ensuring service delivery as time will be allocated to more important farm procedures.

Recently, researchers have conducted a substantial study on livestock projects with a bias towards image acquisition, segmentation/analysis methods, and prediction purposes using either equations/model, or neural networks (NN). On equations, Mutua et al.^9^ obtained length and girth measurements for the prediction of pig weights in rural western Kenya after data was inputted in MS Access and exported to Stata software and subsequently used for statistical analysis. Walugembe et al.^10^ employed prediction equations developed using general linear model procedures to forecast the weights of pigs in Uganda. Using the gig-ethernet camera and multiple slits laser was used by Yoshida et al.^11^ to obtain 3D body measurements in a pig farm. Haq et al.^12^ used multiple linear regression to predict body weights of 521 Jabres cattle in Indonesia using body measurements data. Shuai et al.^13^ collected pig’s body measurements using multiple RGB-D cameras from 3 freely walking pigs.

On NNs, Liu et al.^14^ used an RBF neural network with the growth parameters of 52 Landrace sows for the prediction of pig weights. Salawu et al.^15^ collected parameters (breed, sex, heart girth, length, and height) through a digital weighing scale and applied ANN to predict the body weights of rabbits. Kashiha et al.^16^ utilized the Panasonic WV-BP330 cameras to collect body parameters which were applied in a dynamic data model for weight prediction. Wongsriworaphon, et al.^17^ collected images using Sony DSC-HX5 digital camera and used the vector-quantized temporal associative memory approach, autoregressive and linear embedding models to determine the pigs' bodyweight. Akkol et al.^18^ predicted live weights of hair goats using Artificial Neural Networks (ANNs) and multiple linear regression. Song, et al.^19^ collected morphological traits of dairy cows using a top view 3-D camera (Kinect Sensor for Windows version 2), and thereafter applied a multiple linear regression model for prediction. Pezzuolo et al.^20^ performed weight estimation using multiple linear regression after the collection of requisite images through a Kinect v1 camera. After collecting data using RGB cameras, Yan et al.^21^ utilized edge recognition, and segmentation techniques, to obtain the behinds of pigs in top-view photographs. Then, the data was applied to the Faster-RCNN network.

Our research here aims at building a novel monitoring system with the metrological application of an inexpensive 3-dimensional depth photographic technology to computerize the process of collecting living pigs' physical parameters, allowing for the retrieval of quantitative pig body characteristics, such as body length, height, hip distance, chest girth, back slope, and so on. This is necessary since direct procedures have lots of issues that have been utilized in the past and it entails weighing and conveying the pigs to a weighing station, where they are placed on a digital weighing machine. Specifically, a Multilayer Perceptron (MLP) Neural Network (NN) model and Adam (a replacement optimization algorithm) would also be employed to assess and forecast the pigs' body mass using the measured features.

## Materials and method

Our method herein is divided into two phases i.e. the image acquisition and prediction phase. Between 2016 and 2020, 9980 S21 and S23 Duroc pigs from 3 distinct farms were measured and their body length, height, width, girth, weight, waist, sex, date of birth, and other characteristics were correctly documented. Hundreds of top, side, front, and back-view pictures of pigs in various body positions will be captured for this research. After that, high-quality photos were chosen for further analysis, and the values are utilized for prediction.

### Image acquisition phase

The image acquisition phase commenced with setup for image capturing, computation of length parameters, cloud registration, and target point of cloud removal. The unique approach, which was adapted from Shuai et al.^13^ is built on several relatively less costly but structured Light Depth-Cameras (Microsoft Kinect™ v2), and allows multiple image acquisitions.

### Setup

**Figure 3:**
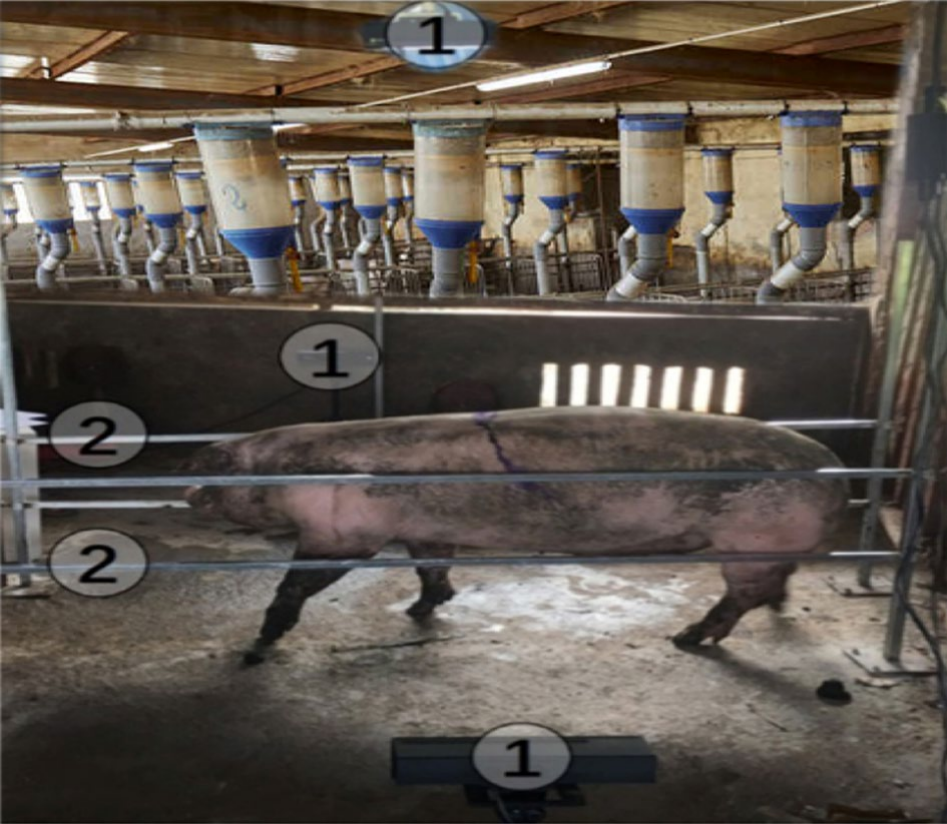
3D data acquisition measurement scene in the Pigpen^13^.

The setup for image acquisition is created in such a manner that walkways’ are provided for the pigs to pass through. Three 3-D cameras (Kinects) positioned on the frame are used to acquire different body postures at different times. A description of the setup alongside a measured pig is depicted in Fig. 3. These cameras from various angles were activated concurrently to capture point clouds (PC) as a pig moved through the viewing platform i.e. the images of the moving pigs are collected in three distinct perspectives (top, left, and right views). The characteristics are acquired, and the spread of the PC is then utilized to determine the measuring points, which enable the accurate measuring of the pig's length, height, abdominal circumference, and breadth. The Kinect v2 was selected as our image collection device because of its motion-tracking capabilities, resolution, and robustness in different illumination conditions. The infrared camera resolution was 512 × 424 pixels, while the other settings are as follows; depth image field of view (70 × 60), measuring range in operation (0.5–4.5 m), as well as frame rate (30 Hz). A microcontroller, which was used for analysis and storage, was attached to every Kinect sensor. The parts of the scene labeled 1 and 2 are the Kinects and railings, respectively.

### Computing the length parameters of living pigs based on the 3D image analysis

The length parameters are consequently extracted from the raw 3D images taken at random by image processing and relevant algorithms. In this stage, the 3D PCs that were initially acquired in the first stage are then preprocessed and registered. Lastly, the attributes of the essential points were discovered and earmarked using our method; thereafter all of the needed features were then measured. The resultant figures are used as inputs at the final stage to predict the actual weight of the pigs. From the capturing system (Kinect V2), the blue part shows the PCs' preprocessing and registration, while the red part indicates the 3D PC collection. The green section is for extracting features and measuring body size.

### Identification and cloud registration/enrollment

**Figure 4:**
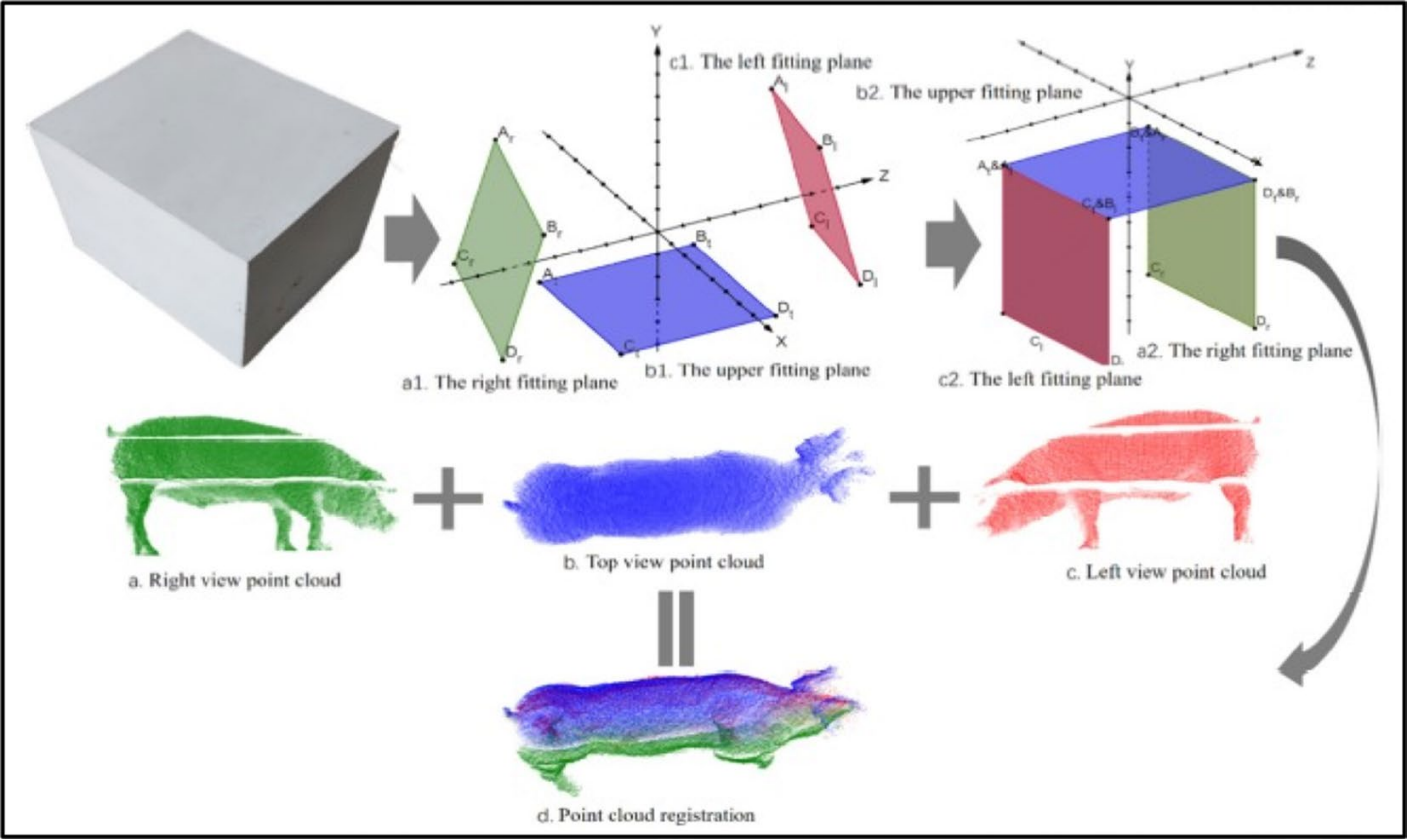
The procedure of PCs enrollment^13^. The registration parameters were obtained and used to reconstruct three local point clouds. Thereafter, the distribution of the point cloud projection in different directions is used to locate the measuring positions, which then contribute to the precise measurement of several key parameters, such as body (length, height, width etc.).

The purpose of PC enrollment is to identify the reconfiguration that successfully allows alignment for all the points in a single coordinate system (CS) given numerous groups of points in various CSs. When it comes to PC registration, there are two main stages: preprocessing/registration and alignment. These steps, which are handled by a PC program, are critical for building noiseless, plain, and detailed PC representations. It is difficult to find the original value used in good registration in the absence of marks, fractions, or geometric features obtained from incorrect enrolment. Enrollment variables were determined with a rectangular cube as a baseline, and cloud points from diverse linkages were combined into a single global integrated platform. Depending on the geometric attributes of the rectangular cube region, multi-view cube PCs are initially discovered whenever the rectangular cuboid was positioned in the finest perspective of the trail. Then, the rotation and translation matrices were computed after every parallel cuboid plane was positioned, together with the geometrical attribute regions. Eventually, enrollment and incorporation of cloud information with multiple views may be accomplished by utilizing the rotation and translation matrices. The entire PC enrolment procedures are shown in Fig. 4.

Figure 3 shows the collection PCs reference, which is mostly a cube, from three various viewpoints and plane-fitting, as well as computing the transformation matrix based on their cube's respective positions. For PC enrollment of pig contours, the transformation matrix is employed. PC enrollment variables generally comprise a rotation matrix (RM) and a translation matrix (TM). RMx, RMy, and RMz are rotating matrices for the directions of x, y, and z-axes, respectively, whereas R is the product of these directions. These matrices and R are formulated as follows:

1 $$RMq = \,\left[ {\begin{array}{*{20}c} 1 & 0 & 0 \\ 0 & {\cos \delta } & { - \sin \delta } \\ 0 & {\sin \delta } & {\cos \delta } \\ \end{array} } \right]$$

2 $$RMr = \,\left[ {\begin{array}{*{20}c} {\cos \varepsilon } & 0 & {\sin \varepsilon } \\ 0 & 1 & 0 \\ { - \sin \varepsilon } & 0 & {\cos \varepsilon } \\ \end{array} } \right]$$

3 $$RMs\, = \,\left[ {\begin{array}{*{20}c} {\cos \lambda } & { - \sin \lambda } & 0 \\ {\sin \lambda } & {\cos \lambda } & 0 \\ 0 & 0 & 1 \\ \end{array} } \right]$$

4 $$R\, = \,RMqRMrRMs$$

where $\delta , \varepsilon , \lambda$ respectively represents the rotating angle axis q, r, s, and R is the function of $Rq,\,Rr,\,Rs.$ We describe point $P(q,r,s)$ in the coordinate system of the data acquisition device and ${P}^{^{\prime}}(q, r, {s}^{^{\prime}})$ in the globally integrated platform. The association between $P(q,r,s)$ and $P^{\prime}(q,r,s^{\prime})$ is based on:

5 $$\left[ {\begin{array}{*{20}c} {q^{^{\prime}} } \\ {r^{^{\prime}} } \\ {s^{^{\prime}} } \\ \end{array} } \right]\,\, = R\left[ {\begin{array}{*{20}c} q \\ r \\ s \\ \end{array} } \right]\, + T\, = \,RMqRMrRMs\,\,\left[ {\begin{array}{*{20}c} q \\ r \\ s \\ \end{array} } \right]\, + \left[ {\begin{array}{*{20}c} {t_q} \\ {t_r} \\ {t_s} \\ \end{array} } \right]\,$$

The parameters $R\, \in \,R^{3 \times 3} \,$ and $T\, \in \,3\, \times \,1$ are $RM$ and $TM$. The $TM\, = \,\left[ {t_q\,t_r\,t_s} \right]^{T}$ indicates the translation range with three axes. More so, the enrollment variables are acquired through a rectangular cube as a reference for powerful durability and high precision.

### Targeted point of cloud removal

Initial cloud data contains targeted pigs to be weighed, railing, concrete laying, and noisy regions. The passed filter is firstly utilized to find PCs within the finest viewing region. Clouds of surplus points mostly envelop the selected pigs and the floor and low clouds are identified using the random sample algorithm (RANSAC). This method was developed based on established threshold factors for the empirical determination of congruent planes. The RANSAC technique for floor plane division efficiently removed the floor points and retrieved the targeted pig points, according to the results. Some noisy points emerge as groupings of nearby points in the retrieved PCs of the pig’s body. However, the Radius Outlier Removal through the filter was used to eliminate the noisy spots. The basic idea is to compute the distance between one point and any of its neighbors, then number how many points are inside the radius r. Supposing, the sum is less than h, the threshold, this position will be labeled an outlier. Following noise removal, PCs of the targeted pigs absent the rails, cement floors, or noise positions were made ready for the subsequent measuring procedure.

### Body size measurement

Retrieved PCs show a variety of orientations and random body positions within the global coordinate system, making it impossible to estimate body sizes instantly. As a result, the topmost viewing PCs were picked manually on the premise that the animals maintain a simple body position. Basic parameters like length, breadth, height, and abdomen all demand to be considered, as they are important parameters for predicting body mass index (BMS) or measuring a pig’s weight^22^. Generally, a tape measure is used to determine the full length of the body beginning with the middle of the linking line across the 2 ears through its earliest natural-born tail root’s wheel down the rear ridge. The gap between the uppermost level of the shoulder and the floor is the height. The abdominal circumference is the perimeter matching the body width region, and this is the largest breadth of the abdomen. Ideally, the target animals stand erect while these body dimensions and features are being measured, as shown in Figs. 1 and 2. Hereunder, the computation of different features using appropriate models was discussed.

Body Length, Height, Width, and Abdominal Circumference**:** The 3 stages involved in measuring length include; aligning the longitudinal segment of the plane (denoted as Z), determining beginning and finishing endpoints, and finding the length of the curve integral. Z is mathematically described as:

6 $$Z\, = \,a0x\, + \,a1y\, + \,a2$$

7 $$S\, = \,\sum\limits_{i = 1}^{n} {(a0xi\, + \,a1yi\, + \,a2\, - \,zi)^{2} }$$

where a_0_, a_1_ a_2_ are the aligning plane variables and S denotes the variation between the clouds of the point $Pcloud\,\,(xi\,yi\,zi)$ from the appropriate plane (Z). As a result, the S ought to be reduced to get a suitable plane. Integration was performed using the PC’s fitting curve on the longitudinal segment.

8 $$L_{length} = \,\int_{xo}^{x1} {\sqrt {(dx)^{2} } } \, + \,(dy)^{2} \, + \,(dz)^{2}$$

Likewise, determining the withers position for the measurement of pig's height is the crucial stage in this approach. The second extreme value position down the vertical axis distribution line is the withers position. The distance between the withers position and the segmentation floor plane may be used to compute height. The pig’s width and abdominal circumference discover the same measurement location i.e. the slice PC of the pig's abdominal region. As a result, the measurement location is selected as the greatest value spanning the frontal and back limbs. However, we identified the hipline and bust position to make it easier to find this spot.

To find the abdominal circumference measurement, the pigs, PCs were taken from the exact measurement region of its width, in addition to that the computation of the circumference of the cut point. Obstacles ought to be taken into account throughout the measuring procedure. For example, the cut PC shows the correctness of the enrollment. Deviation from the enrollment phase will result in the removal of the balance in the abdominal area. Furthermore, the cut PC is hard to bend owing to the railings’ closure and the limit of the Kinect v2’s viewing perspectives. To circumvent these issues, we transferred a cloud of cut spots from Cartesian to Polar coordinates.

The cut point information of the cloud was inserted almost into a round curve, where the shape is equal to the distance from the actual cloud cut. The abdominal band is considerably simpler to compute in the polar CS than in the Cartesian CS since the round curve in the latter is a linear line in the former. Once this line is inserted, it helps to fill in the lost information and improve the variation of the subscription. Additionally, we computed the cut PC's center area and used this as a pole position for establishing a polar link. The shifting relationship between the Cartesian integration system (y, z) and the polar integration system (ρ, θ) are shown as Eqs. (9, 10).

9 $$p_{i} = \sqrt {(yi - y0)^{2} + (zi - z0)^{2} }$$

10 $$\theta_{i} = \left\{ {_{{\tan^{ - 1} \left( { - \frac{{(y_{i} - y_{0} )}}{{(z_{i} - z_{0} )}}} \right) + \frac{3\pi }{2},y_{i} < y_{0} }}^{{\tan^{ - 1} \left( { - \frac{{(y_{i} - y_{0} )}}{{(z_{i} - z_{0} )}}} \right) + \frac{\pi }{2},y_{i} \ge y_{0} }} } \right.$$

Considering the Cartesian coordinate, (yi zi) represents the ith position from the cut layer, while (yo, zo) represents the root location. On the other hand, considering the ith position, P_i_ is the central radius while the θi is the polar angle. Afterward, a smooth, curved block about the size of a cut point was used to mimic the abdominal circumference. This curve was fitted using the most widely accepted kind of b-spline curve^23^. Using the aforementioned approach, the circumference of the abdomen was measured by the model below.

11 $$S = \mathop \int \nolimits_{0}^{{2\pi }} \sqrt {\left( {f(\theta )} \right)^{2} + (\mathop f\limits^{{\hat{A}^{\prime}}} (\theta ))^{2} d\theta }$$

where f(θ) denotes the function θ ∈ (0, 2π) based on the curve that fits and f (θ)$\mathop f\limits^{{\,\,\,\,\,{{\hat{\rm A}^{\prime}}}}} (\theta )$ depicts the function’s value i.e. f (θ).

### Ethics declaration

1. Measurements were done in accordance with guidelines of Institutional Animal Care and Use Committee of South China Agricultural University (Guangzhou, People’s Republic of China).
2. The experimental procedures were done in line with approval of Institutional Animal Care and Use Committee of South China Agricultural University (Guangzhou, People’s Republic of China).
3. All animals used in this study were properly managed by the handlers with utmost care to avoid stress.
4. The measurements of the pigs were done and displayed in our methodology to show the reliability of our study.
5. Figures 1 and 4 showed individuals involved during the manual measurements of the pigs, and we state that the displayed pictures with human face can be published online as adequate clarifications were sought.
6. A clear consent was obtained from the handlers that assisted us during our morphometric measurements and they have agreed that their images can be published online.

## The proposed approach

### The MLP NN models

**Figure 5:**
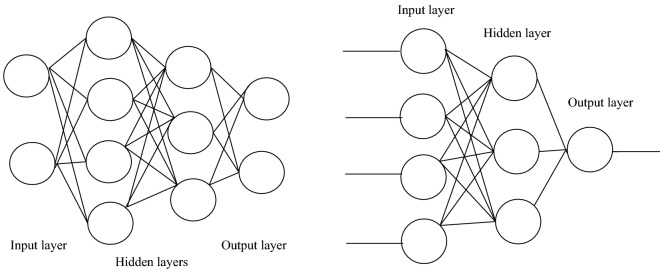
Different MLP representations showing the layers^27,28^.

**Figure 6:**
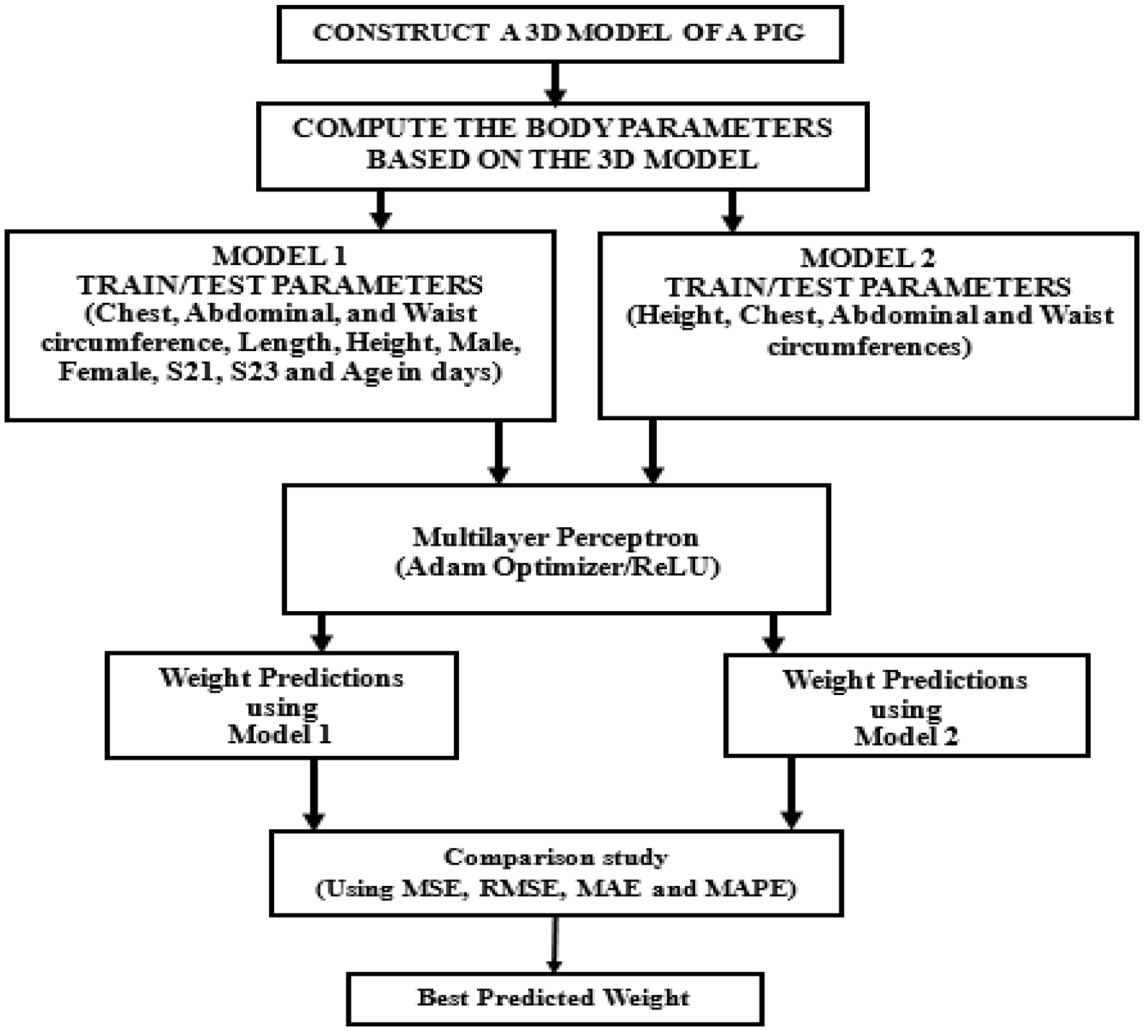
Framework for MLP implementation and evaluation.

This phase includes the activities listed as follows: preprocessing of the acquired data; regression and prediction of body weight using supervised learning algorithms. Here, MLP is the proposed Neural Network (NN) model with other related algorithms, which are used to predict the actual weight of the pigs at varying periods. MLP is a machine learning (ML) classification technique based on feedforward neural networks (FFNNs), which are composed of ordered layers comparable to human neuron processing^24^. It is comprised of many neurons that serve as processing components and are arranged in a sequence of completely linked stacked layers. As succinctly defined by Laudani, et al.^25^, the internal architectural framework of an FFNN is structured in such a manner that successive layers of neurons and interconnections are created using the following guidelines: Each layer's neuron is linked to all (and solely) the next layer neurons. MLP is a special category of FFNN^26^. The diagrams in Fig. 5 represents the schematic representation of MLP^27^ (Deyasi), while the one on the right represents a MLP model with one hidden layer^28^ (Carlson). These diagrams clearly illustrate a collection of hidden layer inputs, which are trained to produce the expected result, which in our context is the pig's weight. In the same vein, the proposed MLP has a single input layer, three hidden layers, and one output layer. This input layer holds different sets of input at every particular instance representing different parameters i.e. length, width, and height, etc. Note that the first hidden layer has 5 neurons, while the second and third layers have 4 neurons each. However, the flow of activities required for MLP implementation and evaluation is represented in Fig. 6.

For the predictive modeling of pig weights using MLP, libraries such as PyTorch^29^ and Scikitlearn^30^ were imported on Google Colab. In the same environment, libraries such as Linear, Rectified Linear Unit (ReLU), Adam, MSELoss were also activated for linear regression modeling. Then Seaborn^31^ and Matplotlib^32^ were imported for visualization. Note that the Adam optimizer algorithm was used because it is a straightforward and computationally effective strategy for gradient-based optimizations. It is also used to change the attributes of the NN, thereby reducing losses. This chosen optimizer incorporates the benefits of two prominent optimization strategies that have lately become popular i.e. AdaGrad and RMSProp. While the former can handle sparse gradients, the latter can effectively manage non-stationary purposes. The approach is simple to execute and consumes minimal storage^33^. At each node in the model, an activation function, ReLU was used to transform the weighted sum of the input into an output from a node(s) in the hidden layers of the network and is given by y = max (0, x). For the correctness and accuracy of the results, backpropagation was employed by continuously adjusting the weights to achieve a better output.

### Dataset

**Figure 7:**
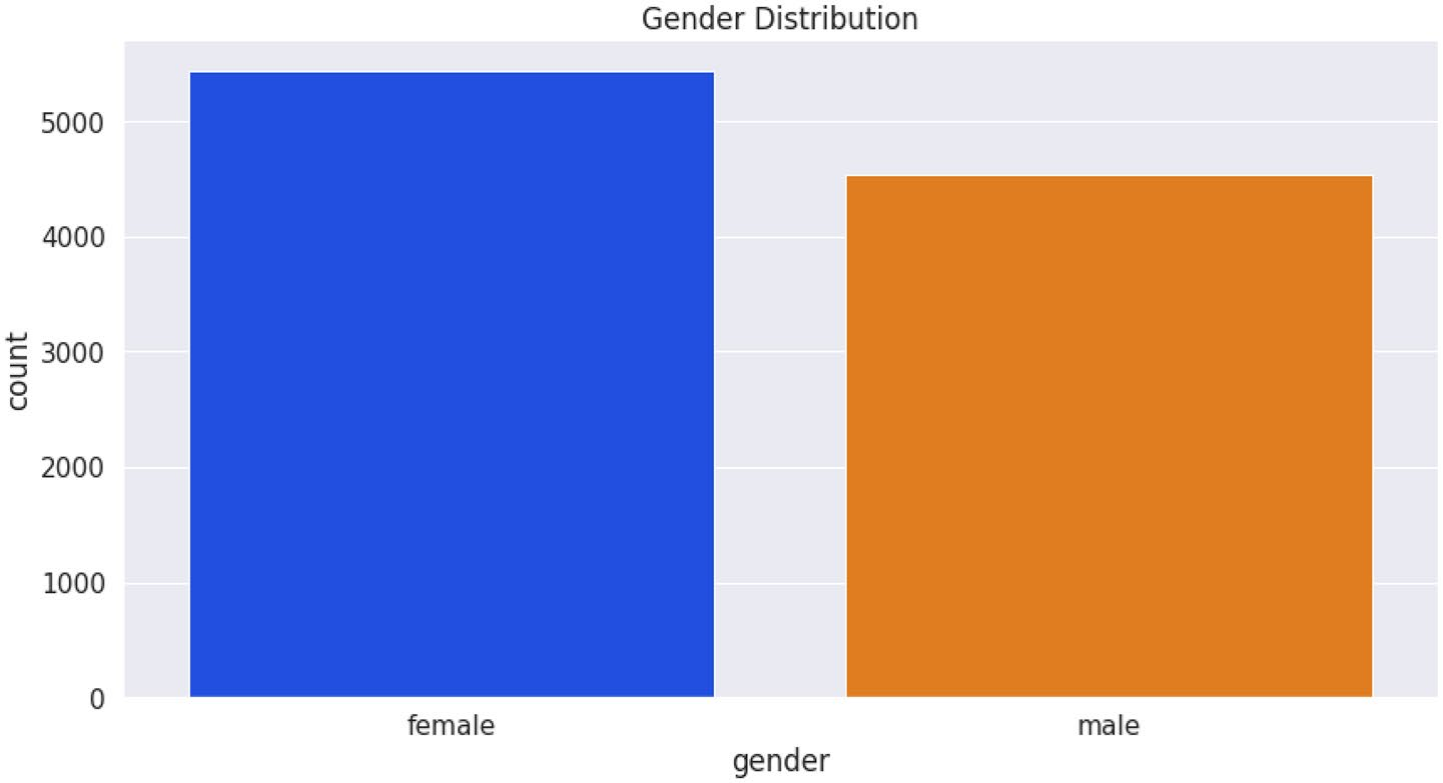
Gender distribution from the dataset.

**Figure 8:**
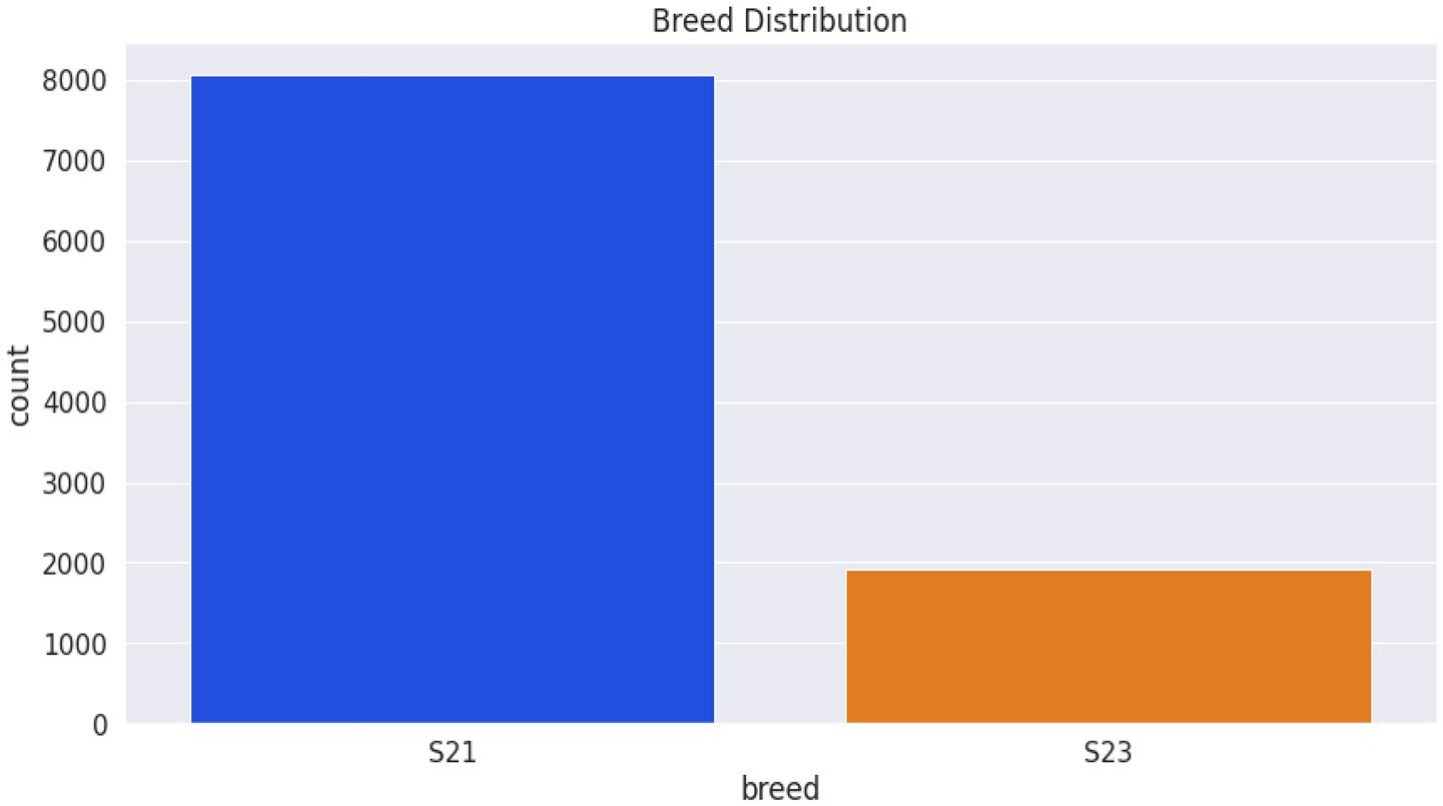
Breed distribution from the dataset.

**Figure 9:**
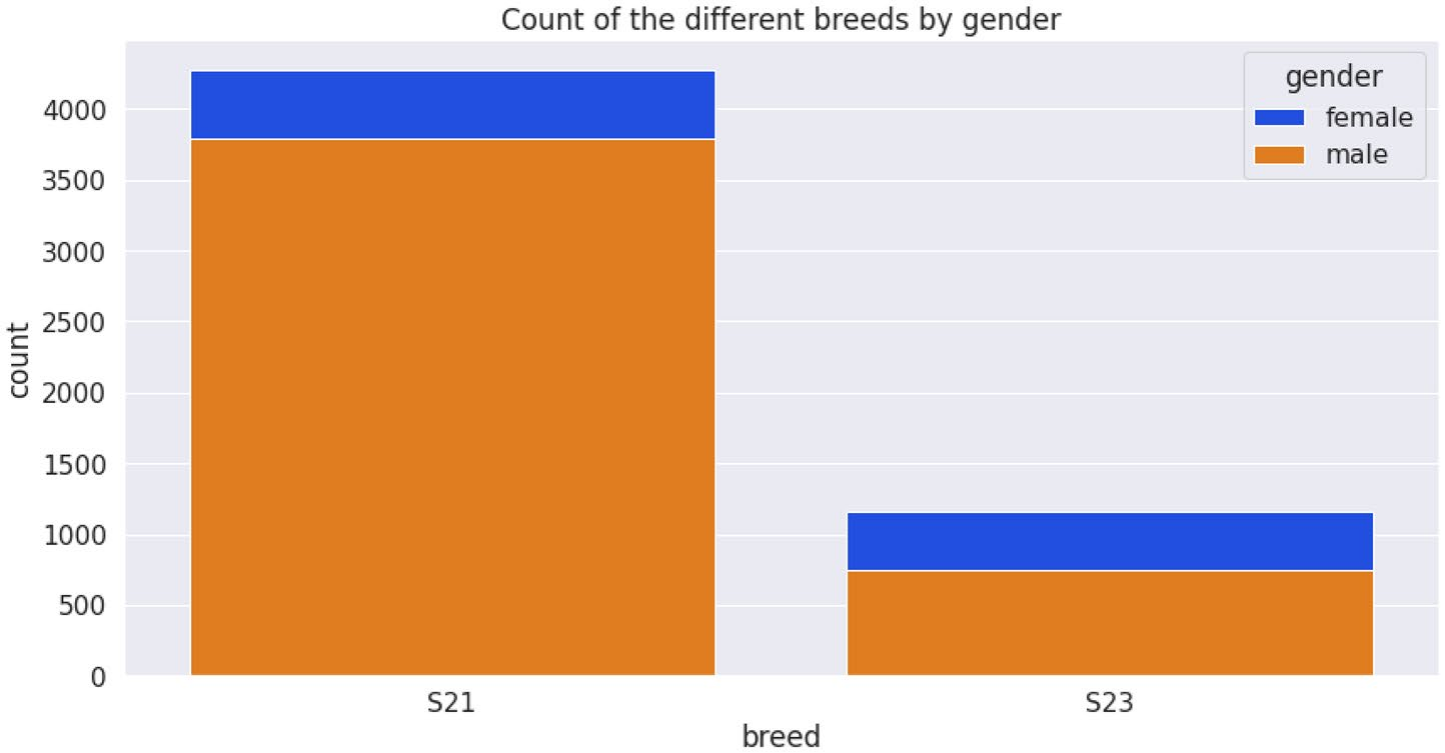
Distribution of different breeds by gender.

**Figure 10:**
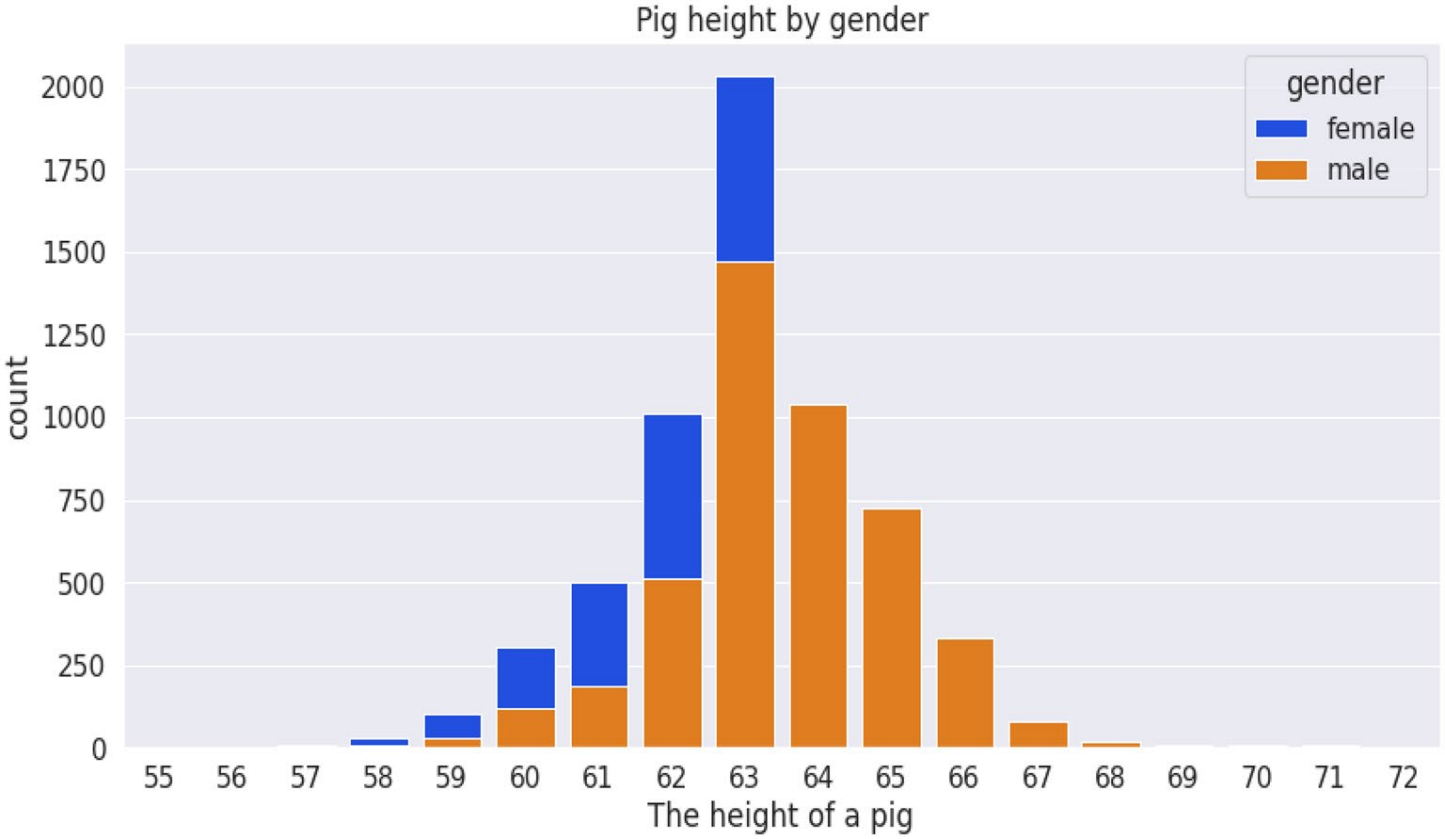
Distribution of heights by gender.

The dataset was saved on a computer as an excel sheet and then read in CSV format. Collected data were saved under the following column headings: breed, gender, date of determination, chest circumference, abdominal circumference, waist circumference, date of birth, weight measurement, length, and height. The female (blue) and male (yellow) are 5438 and 4542 in number (Fig. 7) while the S21 and S23 breeds are 8068 and 1912 respectively (Fig. 8). The average weight measurements of S21 and S23 breeds are 111.71 and 105.87 while that of the females and males are 108.46 and 113.14, respectively. Figure 9 depicts the distribution of different breeds by gender, whereas Fig. 10 depicts the distribution of heights by gender. The dataset showed an intense need for data cleaning. Therefore, to implement this we did the following: created a new column called age in days, which is the difference between the determination date and birth date, converted the gender and breed types to numerical variables (OneHot encoding), and then the addition of missing values in the pig height using the median height. The result of the actual data used is contained in Table 1, which shows the first five records of cleaned data. Additionally, the dataset was normalized using the sklearn.preprocessing package and Table 2 shows the first five records of normalized data.

**Table 1:** Dataset sample.

Chest	Abdomen	Waist	Length	Height	Female	Male	S21	S23	Age	Weight
110.0	115.0	107.0	120.0	63.0	1.0	0.0	1.0	0.0	193.0	113.5
105.0	116.0	106.0	119.0	63.0	1.0	0.0	1.0	0.0	192.0	111.4
109.0	120.0	104.0	125.0	63.0	1.0	0.0	1.0	0.0	194.0	114.6
105.0	112.0	100.0	123.0	63.0	1.0	0.0	1.0	0.0	192.0	111.8
107.0	116.0	106.0	118.0	63.0	1.0	0.0	1.0	0.0	189.0	114.2

**Table 2:** Normalized dataset sample.

Chest	Abdomen	Waist	Length	Height	Female	Male	S21	S23	Age
0.3390	0.3544	0.3298	0.3698	0.1942	0.0031	0.0000	0.0031	0.0000	0.5948
0.3270	0.3612	0.3301	0.3706	0.1962	0.0031	0.0000	0.0031	0.0000	0.5979
0.3325	0.3660	0.3173	0.3813	0.1922	0.0031	0.0000	0.0031	0.0000	0.5918
0.3287	0.3506	0.3131	0.3851	0.1972	0.0031	0.0000	0.0031	0.0000	0.6011
0.3337	0.3618	0.3306	0.3680	0.1965	0.0031	0.0000	0.0031	0.0000	0.5895

### Model construction

Two models (Model 1 and 2) were initially developed and used for the prediction, to find out the model with the best performance when checked with some evaluation metrics. In other words, the study is a quest to find out if lesser parameters can give similar result/accuracy compared to prediction using all the features. Here, the hypothesis goes thus: there is no significant difference between predictions using 4or less correlated features and predictions using all collected features of pigs. After normalization, this hypothesis is further extended to accommodate results of performing prediction using 3 and 2 features, and evaluating if there is any significant difference with weight prediction using all the pigs’ features.

Specifically, model 1 contained all the features i.e. length, height, male, female, S21, S23, age in days, chest, abdominal, and waist circumferences. Weight measurement was used as the label. On the other hand, model 2 construction only involved chest, abdominal, waist circumferences and their height measurements. These features were selected based on correlation analysis of the entire feature set and they showed high correlation leading to multicollinearity. Models 3 and 4 were subsequently developed (with normalized dataset) and also used for prediction. The results shown in Table 6 confirm the importance of normalization as indicated by the superior and less RMSE values. Also, we ascertained that the body weight could be predicted using less number of correlated features as seen in Model 3 which utilized 2 features for the prediction. Table 3 shows the matrix (symmetrical) of correlation between each pair of features.

**Table 3:** Correlation matrix between each pair of features.

	Chest	Abdominal	Waist	Length	Height	Female	Male	S21	S23	Age in days	Weight
Chest	1.0000	0.8323	0.8571	0.5557	0.4436	− 0.2063	0.2063	0.1743	− 0.1743	− 0.0525	0.6434
Abdominal	0.8323	1.0000	0.8276	0.5607	0.4477	− 0.2009	0.2009	0.2111	− 0.2111	− 0.0913	0.6152
Waist	0.8571	0.8276	1.0000	0.5293	0.4118	− 0.1949	0.1949	0.2011	− 0.2011	− 0.0792	0.6056
Length	0.5557	0.5607	0.5293	1.0000	0.5912	− 0.2971	0.2971	0.2334	− 0.2334	− 0.0294	0.6921
Height	0.4436	0.4477	0.4118	0.5912	1.0000	− 0.2579	0.2579	0.1487	− 0.1487	− 0.0350	0.4332
Female	− 0.2063	− 0.2009	− 0.1949	− 0.2971	− 0.2579	1.0000	− 1.0000	− 0.0609	0.0609	0.2016	− 0.3313
Male	0.2063	0.2009	0.1949	0.2971	0.2579	− 1.0000	1.0000	0.0609	− 0.0609	− 0.2016	0.3313
S21	0.1743	0.2111	0.2011	0.2334	0.1487	− 0.0609	0.0609	1.0000	− 1.0000	− 0.3219	0.3260
S23	− 0.1743	− 0.2111	− 0.2011	− 0.2334	− 0.1487	0.0609	− 0.06093	− 1.0000	1.0000	0.3219	− 0.3260
Age in days	− 0.0525	− 0.0913	− 0.0793	− 0.0294	− 0.0349	0.2016	− 0.2016	− 0.3219	0.3219	1.0000	0.0794
Weight	0.6434	0.6152	0.6056	0.6921	0.4332	− 0.3313	0.3313	0.3260	− 0.3260	0.0794	1.0000

### Model training and testing

The dataset was split into training, testing and validation sets: 70 percent of the dataset was used to train the linear regression model and 15 percent each were used for testing and validation purposes. Then the individual datasets are then converted into tensors to be utilized by the Pytorch Framework^29^. Model 1 and model 2 were trained for 300 epochs, batch sizes of 10, and a learning rate of 0.01. The gradients were cleared using the optimizer. The losses are computed using MSELoss to display corresponding outputs, and this MSELoss referred to as the mean squared error, is also utilized as the metric of evaluation. Also, training and validation losses constitute our outputs in the next section. Note that the training loss is a statistic that measures how well a MLP model matches the training set. Validation loss, on the other hand, is a measure used to evaluate the effectiveness of a MLP model performance on the validation data set.

## Results

**Figure 11:**
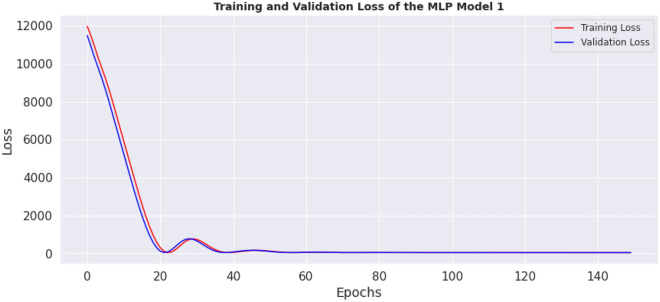
Loss of MLP Model 1.

**Figure 12:**
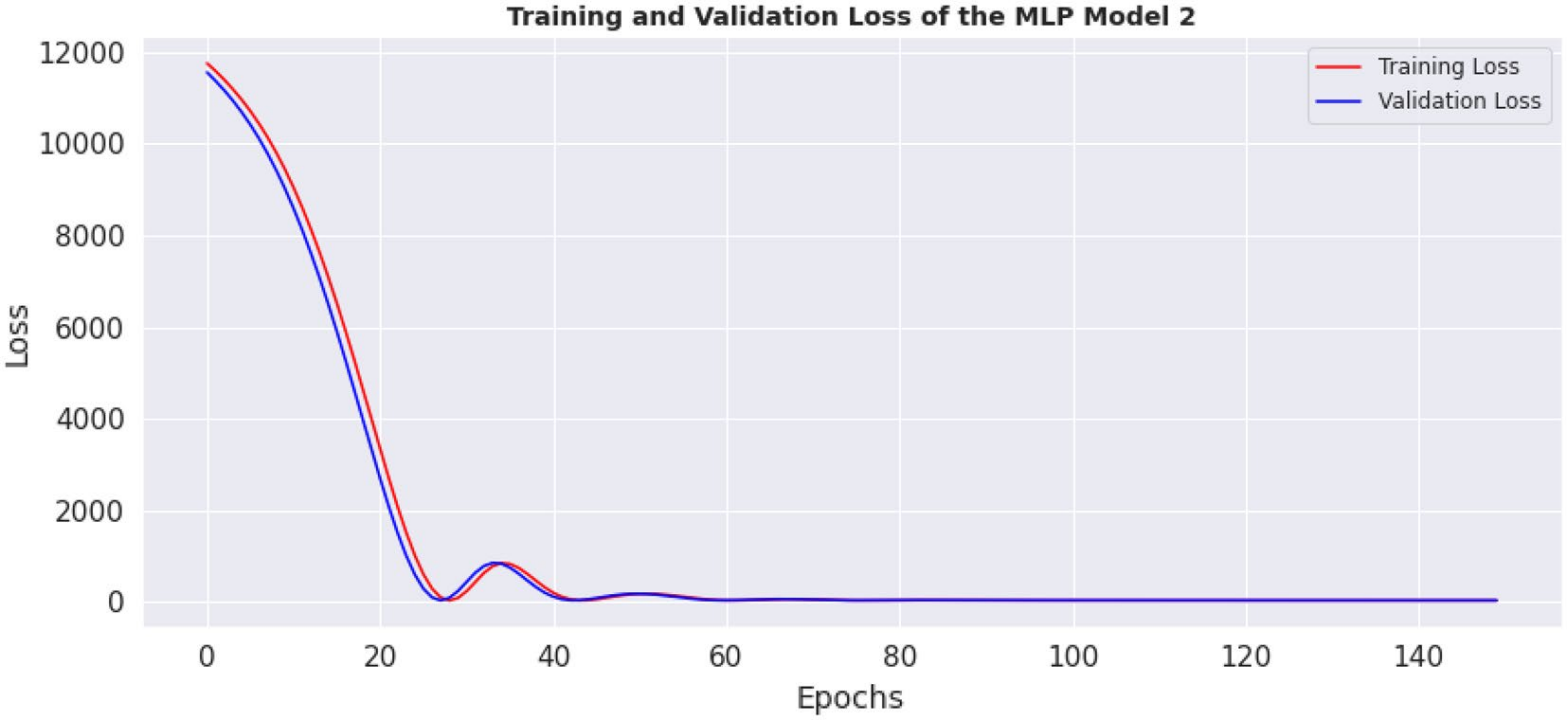
Loss of MLP Model 2.

This section contains the actual evaluation i.e. the prediction and comparative analyses. The loss of MLP model 1 is shown in Fig. 11, while the loss of MLP model 2 is shown in Fig. 12. Reviewing the diagrams, one can easily observe some interesting patterns. From both figures it could be observed that, the models converge after the 100th epoch. However the graphs show the losses up to the 150th epoch.

In other to compare actual and predicted weights, MSE and RMSE were used for evaluation. MSE evaluates the average squared difference between the observed and predicted values^34^. RMSE is denoted as the square root of the mean of the square of all of the error^35^ or “the standard deviation of the residuals or prediction errors”^36^. Other evaluation metrics used in the study are the mean absolute error (MAE) and the mean absolute percentage error (MAPE). The formulas of metrics are as follows:

$$\begin{gathered} MSE\, = \,\,\frac{1}{N}\,\sum\limits_{i = 1}^{N} {(Actuali\, - \,Predictedi)^{2} } \hfill \\ RMSE\, = \,\sqrt {\frac{1}{N}\,\sum\limits_{i = 1}^{N} {(Actuali\, - \,Predictedi)^{2} } } \, \hfill \\ MAE\, = \,\,\frac{1}{N}\sum\limits_{i = 1}^{N} | Actuali\, - \,Predictedi| \hfill \\ MAPE\, = \,\,\frac{1}{N}\,\sum\limits_{i = 1}^{N} {|\frac{Actuali\, - \,Predictedi}{{Actuali}}} | \hfill \\ \end{gathered}$$

**Figure 13:**
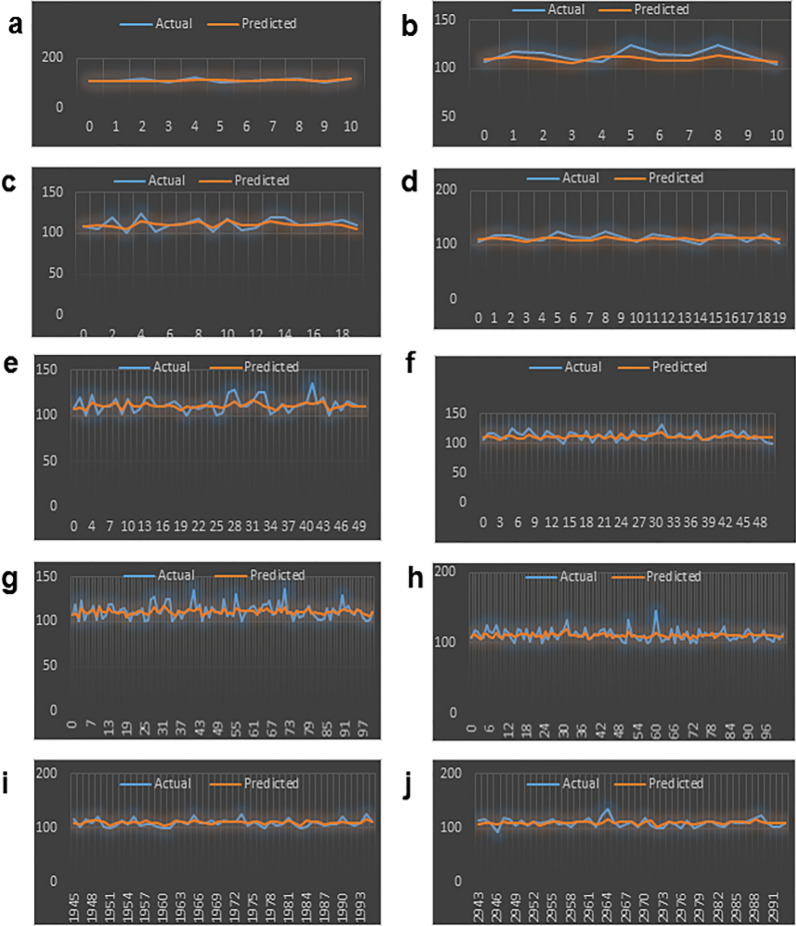
Model 1’s and Model 2’s Actual and Predicted Values in (**a**, **b**) for 1st 10 Records; (**c**, **d**) for 1st 20 Records; (**e**, **f**) 1st 50 Records; (**g**, **h**) for 1st 100 Records; and (**i**, **j**) for last 50 Records, respectively.

During predictions, model 1 generated a total record of 1995 while 2993 records were later generated for model 2. Model 1 and model 2 have RMSE values of 5.5758 and 6.0407, respectively, which imply that Model 1 performed better since its RMSE value is lower. Other metrics used are the mean absolute error (MAE) and the mean absolute percentage error (MAPE). As shown in Table 4 below, model 1 has the least of the errors for all the evaluation metrics i.e. the errors incurred by model 1 for the MSE, RMSE, MAE and the MAPE are the least between the two models. Conclusively, model 1 fits the dataset better hence the better model for predicting the weight of the pigs. Some of the prediction records showing the differences and variations in both models are plotted and shown as follows. Figure 13a, b shows the first 10 records of models 1 and 2’s actual and predicted values; (c, d) showed the first 20 records of same; (e, f) shows the first 50 records; (g, h) shows the first 100 records; while the last 50 records of models 1and 2’s actual and predicted values were shown in (i, j).

**Table 4:** Evaluation metrics for the two MLP models.

S/N	Metrics	Model 1	Model 2
1	MSE	31.0890	36.4906
2	RMSE	5.5758	6.0407
3	MAE	4.4420	4.7774
4	MAPE	0.03991	0.0429

### Impact of normalization

**Figure 14:**
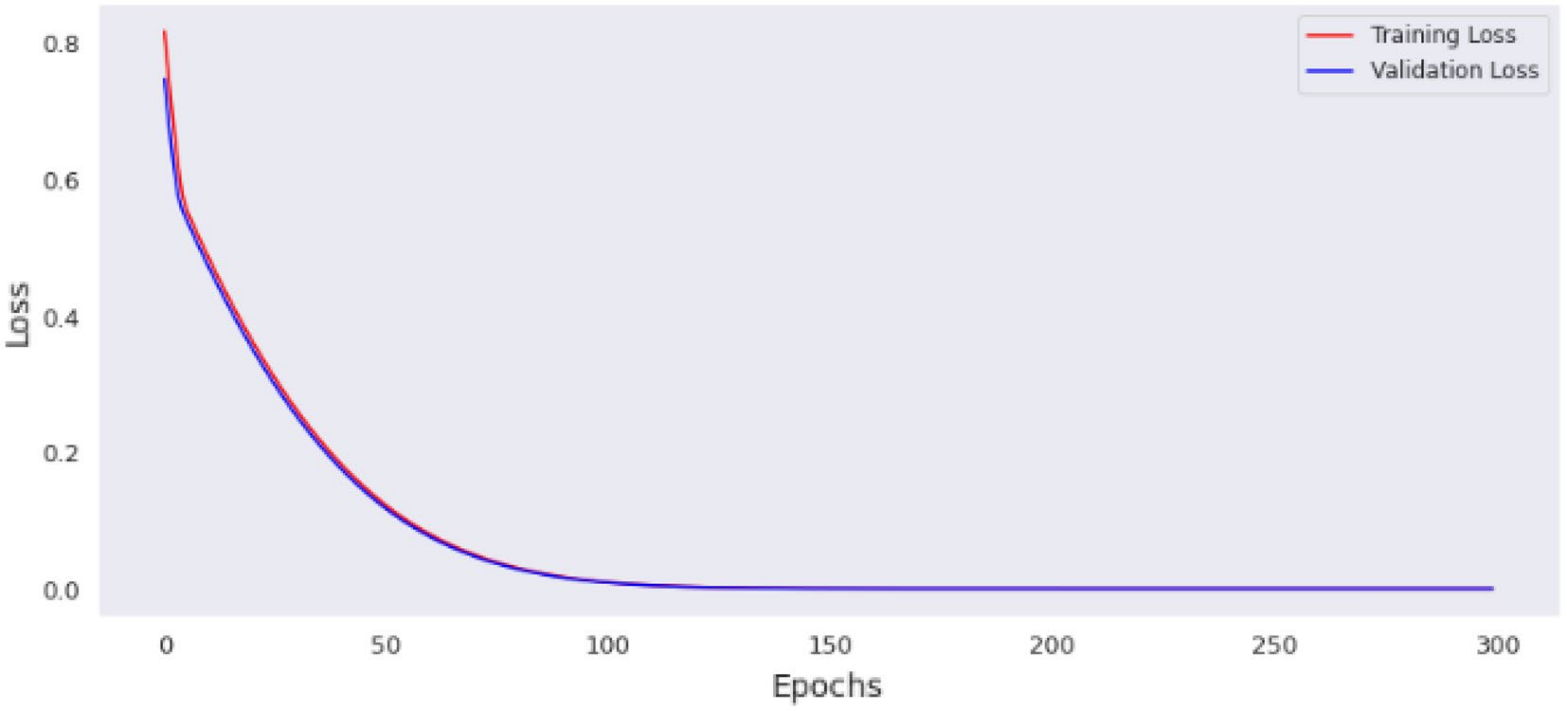
Loss of MLP Model 1 using normalized dataset.

**Figure 15:**
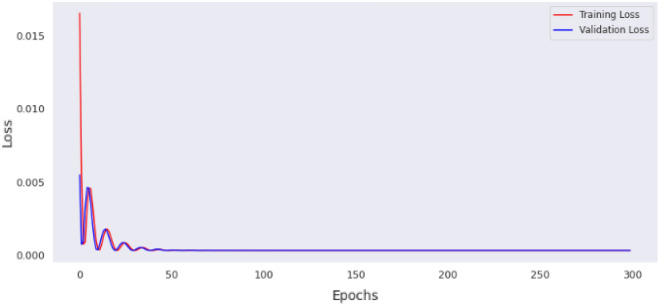
Loss of MLP Model 2 using normalized dataset.

**Figure 16:**
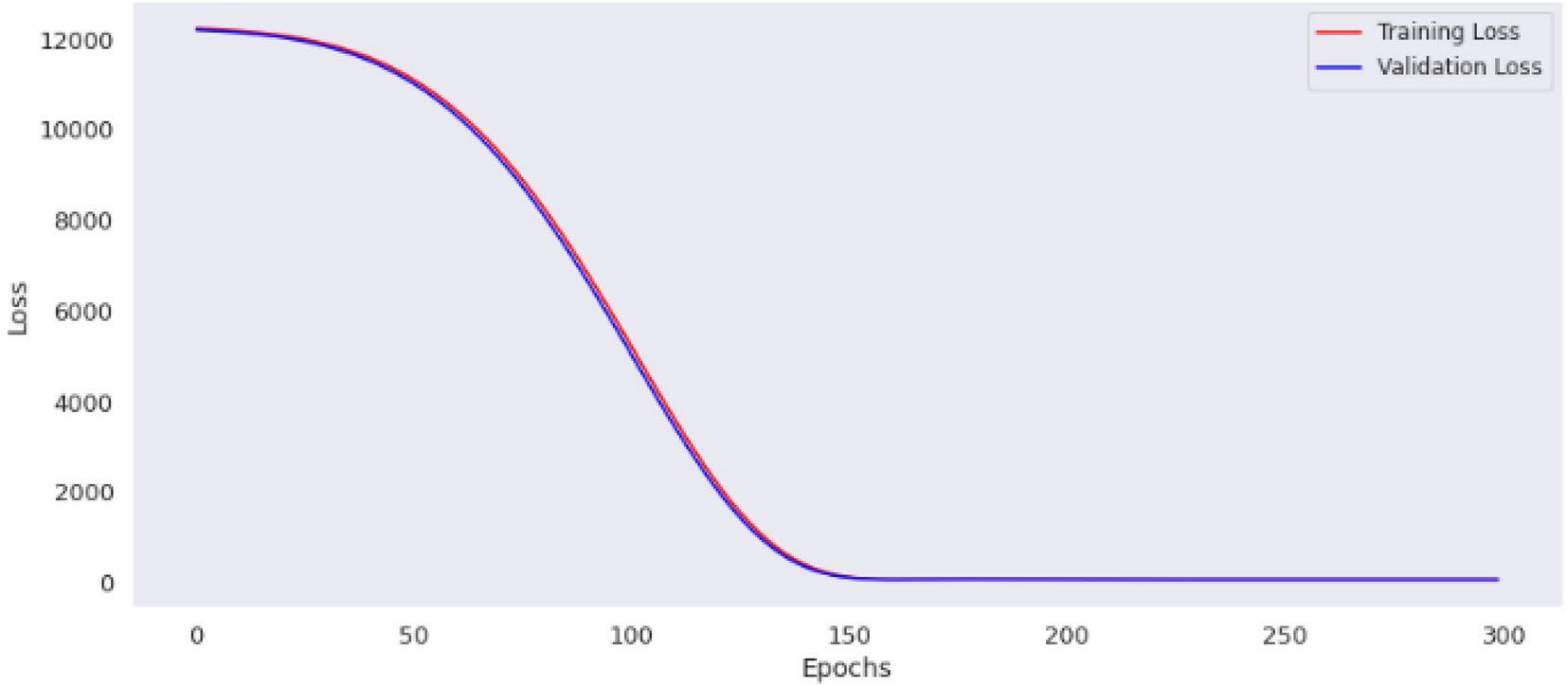
Loss of MLP Model 3 using normalized dataset.

**Figure 17:**
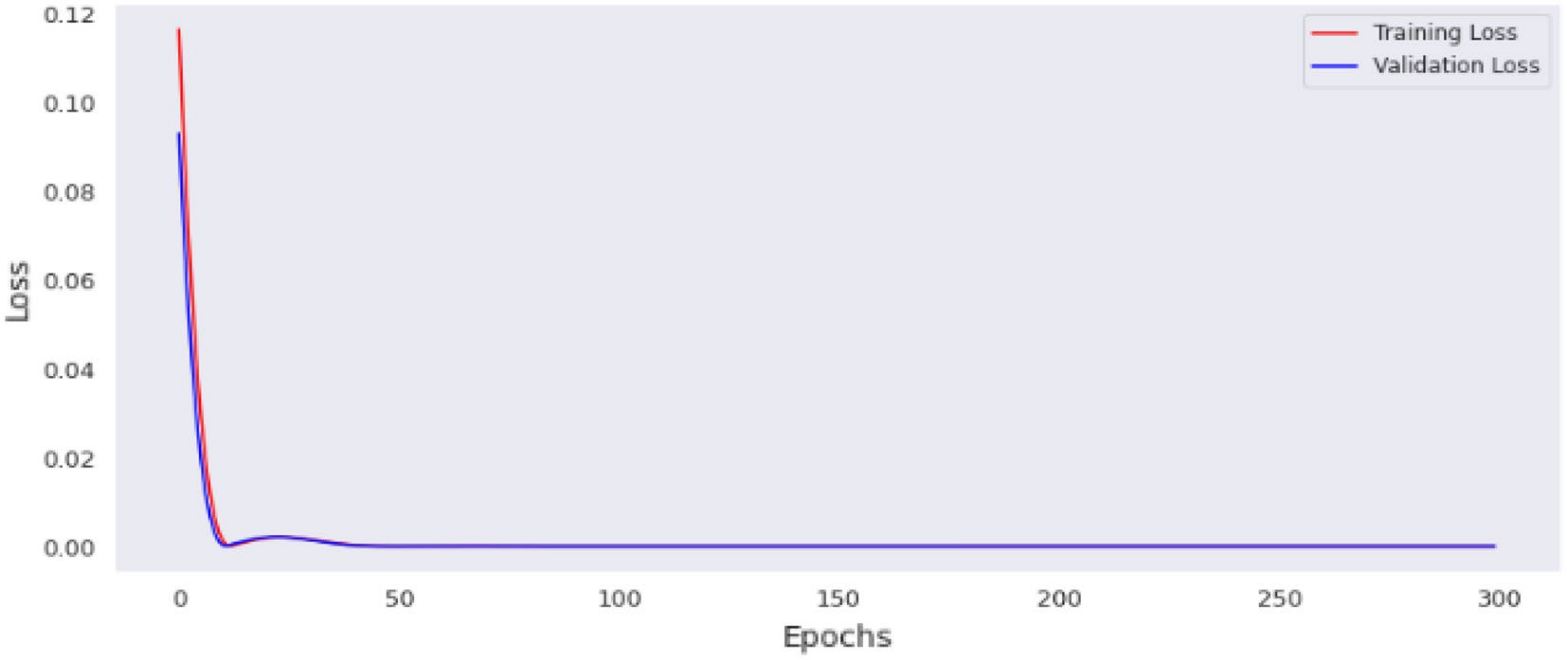
Loss of MLP Model 4 using normalized dataset.

The preprocessing library was imported from Scikitlearn^30^ and the normalize method used for the normalization of the dataset. Normalization additionally renders the training procedure less responsive to feature scale. As a consequence, following training, the coefficients are improved. Besides normalization, we also explored the case of selecting variables for newer models using a different method, wherein variance of each feature is estimated and those with low variance were excluded based on a chosen threshold. We found the variance of the normalized dataset (Table 5) and then evaluated the outcomes of 0.00006 and 0.00007 thresholds. At 0.00006 (threshold 1), the remaining variables include age, length, chest, abdominal and waist circumferences, thus constituting the variables for model 3. While at 0.00007 (threshold 2), the remaining variable are age (in days) and the abdominal circumference and these constituted model 4. For each case, the variables that were not selected are of course below these chosen thresholds. Losses incurred as result of training the normalized data were plotted for the four models and depicted as Figs. 14, 15, 16 and 17. From these figures, one can conclude that both the training and validation losses both reduce and stabilize at a certain point. Table 6 below contains the results of assessing the performance of the four models using MSE, RMSE, MAE and MAPE.

**Table 5:** Variances of the pig’s variables.

S/N	Metrics	Variance
1	Chest circumference	0.000061
2	Abdominal circumference	0.000084
3	Waist circumference	0.000070
5	Length of pig	0.000067
6	Height	0.000030
7	Female	0.000003
8	Male	0.000003
9	S21	0.000002
10	S23	0.000002
11	Age in days	0.000484

**Table 6:** Evaluation metrics for MLP models with normalized dataset.

S/N	Metrics	Model 1 (All variables)	Model 2 (Height, chest, abdominal and waist circumferences)	Model 3 (Abdominal circumference, and age)	Model 4 (Length, age, chest, abdominal, and waist circumferences)
1	MSE	48.4219	28.0023	28.0023	28.0023
2	RMSE	6.9586	5.2917	5.2917	5.2917
3	MAE	5.4150	4.1577	4.1577	4.1577
4	MAPE	0.0486	0.0375	0.0375	0.0375

With the normalized dataset, the evaluation metrics showed best results with model 2, model 3 and model 4. Specifically, model 2 involved 4 features namely, height, chest, abdominal and waist circumferences; model 3 involved two features namely, abdominal circumference, and age, while model 4 involved length, age, chest, abdominal, and waist circumferences. It is worthy of note that for the case where the dataset was not normalized, model 1 with least values for all metrics gave the best result. This clearly shows that working with un-normalized dataset can mislead the modeler or analyst. Models 2, 3 and 4 have the same MSE, RMSE, MAE and MAPE values, thus outperforming Model 1.

## Conclusion

In this study, we constructed a 3D model of a pig and developed MLP NN models, which are a feed-forward ANN. The study incorporated the Adam optimizer and the ReLU activation functions as well as other algorithms towards obtaining good results. This independent system would eliminate internal and external biases prevalent in body weight measurement and ultimately help for optimal data collection and interpretation. Our choice of method is ideal for ML challenges with datasets. This prediction approach was applied after body parameters such as length, height, width etc. were obtained using light depth-cameras (Microsoft Kinect™ v2). The data automatically acquired would assist to effectively manage and minimize the cost of pigs’ production and welfare since weight overestimation and underestimation were eliminated. At first, two MLP models were developed and trained, with an un-normalized dataset; analyses showed that model 1 performed better than model 2 in the prediction of pig weights with a RMSE value of 5.5 and 6.0 respectively. Furthermore, two additional models (3 and 4) were developed and trained alongside with the first two models using normalized dataset. Results showed that models 2, 3 and 4 performed better with RMSE values of 5.29 than model 1with a RMSE value of 6.95 as shown in Table 6. Model 3 generated an interesting discovery, which is the accurate prediction of pigs’ weights using only two features i.e. abdominal circumference and age. The study avails a method to automatically and efficiently compute the bodyweight of live pigs using other body character traits at various phases of their development while buttressing the importance of using normalized datasets in regression analyses. It is noteworthy that, due to the decrease in feed costs which contributes for above 60% of operational expenses when precisely predicted, and accuracy in projecting pig weights contributes to profits in both private and commercial farms. Furthermore, the research protects pigs from infections and injuries caused by manual data collection processes. In the future, pig images and dataset will be used to explore the concept of generative adversarial networks^37,38^.

## Data Availability

The datasets used and/or analyzed during the current study available from the corresponding author on reasonable request.
